# Information experiences among women diagnosed with endometrial cancer: a mixed methods study

**DOI:** 10.1007/s00520-026-11082-2

**Published:** 2026-08-11

**Authors:** Tracey DiSipio, Britta Wigginton, Joan Cunningham, Bena Brown, Susan Jordan, Abbey Diaz

**Affiliations:** 1 School of Public Health, The University of Queensland, 288 Herston Road, Brisbane, Queensland Australia; 2 Menzies School of Health Research, Charles Darwin University, Darwin, Northern Territory Australia; 3 Southern Queensland Centre of Excellence in Aboriginal and Torres Strait Islander Primary Health Care, Metro South Health, Inala, Australia; 4 Yardhura Walani National Centre for Aboriginal and Torres Strait Islander Wellbeing Research, National Centre for Epidemiology and Population Health, Australian National University, Canberra, Australia

**Keywords:** Endometrial cancer, Information needs, Information satisfaction, Journey mapping, Mixed methods

## Abstract

**Purpose:**

Despite a growing population of endometrial cancer survivors, there is limited evidence about their supportive care needs. Unmet information needs are commonly reported throughout the cancer journey. This study examined information needs, satisfaction, and sources of information among women recently diagnosed with endometrial cancer at key touchpoints: pre-diagnosis, diagnosis, and treatment.

**Methods:**

This cross-sectional mixed methods study recruited women and people with female reproductive organs identified via the Queensland Cancer Register who were diagnosed in the previous 13 months. Self-reported questionnaires were administered and analyzed descriptively. We interviewed a subset of women and analyzed these data using content analysis. Journey mapping was used to describe information experiences across key touchpoints.

**Results:**

Eighty-two women completed a questionnaire and 24 were also interviewed. We found moderate-to-high unmet information needs during pre-diagnosis (58%), diagnosis (71%), and treatment (57%). Satisfaction with the type and timing of information was lowest pre-diagnosis and improved during later touchpoints. Doctors, internet searches, family/friends, and printed materials were key information sources. Qualitative themes highlighted individual variation in information needs, shaped by cognitive and emotional capacity. Women valued clear, compassionate information delivered progressively.

**Conclusion:**

Many women experienced delayed and inconsistent communication about their cancer diagnosis and treatment. Healthcare professionals play a central role in information delivery and should offer clear, timely, and respectful support across the care continuum that is tailored to women’s needs and capacities. Addressing unmet information needs at key points may enhance patient experience and enable informed decision-making.

**Supplementary Information:**

The online version contains supplementary material available at https://doi.org/10.1007/s00520-026-11082-2.

## Introduction

Endometrial cancer, originating in the epithelial lining of the uterus (endometrium), accounts for 90–95% of uterine cancer cases [1]. Incidence has risen over the past two decades [2–5], partly due to the ageing population and increasing incidence of obesity [6]. Although there is no effective screening, most cases are diagnosed early, with 5-year survival exceeding 80% in Western countries such as the USA and Australia [5, 7].

Meeting patient information needs is fundamental to support them across the cancer care continuum [8]. High-quality health information improves knowledge, supports decision-making, and enhances care experiences [9]. Evidence across cancer types demonstrates that meeting information needs before and after treatment benefits patients’ psychological well-being, while unmet needs increase anxiety and depression and reduce quality of life [10–12]. Information needs also vary by age, cancer type, time since diagnosis, treatment modality, and coping style [13].

However, little is known about the information needs of Australian women and people with female reproductive organs^1^ diagnosed with endometrial cancer. While symptoms of abnormal gynecological bleeding may prompt people to consult a healthcare professional and then assessed for endometrial cancer, this is not always the case [14]. Barriers in receiving a timely diagnosis have been reported in international literature, including women’s limited knowledge about gynecological health, lack of awareness of endometrial cancer, gaps in health literacy, healthcare professionals being unfamiliar with symptoms, and structural and communication barriers [15, 16]. European cohorts further highlight dissatisfaction with the information received about diagnosis and treatment [17, 18].

Understanding the current information landscape may help identify points along the cancer trajectory where there are high needs for better information delivery. The Model of Pathways to Treatment [19] describes the events (e.g., detection of bodily changes; first consultation with a healthcare professional) and processes (e.g., patient appraisal and self-management; healthcare professional appraisal, investigations, and referrals) leading to cancer treatment, informing the key touchpoints for this study: (1) pre-diagnosis, (2) diagnosis, and (3) treatment. These touchpoints refer to specific times where a person interacts with the healthcare system. The aim of this mixed methods study was to investigate and describe the information experiences of people diagnosed with endometrial cancer at each key touchpoint. The objectives were to investigate information experiences including information needs, information satisfaction, and preferred sources of information.

## Materials and methods

### Study design

This cross-sectional mixed methods study included a mailed self-administered questionnaire and subsequent semi-structured telephone interviews with women diagnosed with endometrial cancer in Queensland, Australia. Information from the Queensland Cancer Register (QCR) (i.e., age at diagnosis, stage of disease, remoteness of residence [20], socio-economic status [21], First Nations status) was also available. Ethics approval was obtained prior to study commencement from The University of Queensland Human Research Ethics Committee (HE000046). The Mixed Methods Reporting in Rehabilitation and Health Sciences (MMR-RHS) checklist was followed [22].

### Participants

Cancer Alliance Queensland (CAQ), which houses the QCR, was used to identify potential participants. All endometrial cancer notifications received by the QCR in the 13 months prior to recruitment (01/08/2022 to 31/08/2023) were identified. Eligible women were 18+ years, diagnosed with primary endometrial cancer (ICD-10 C54.1), and living in Queensland at the time of diagnosis. Cases aged over 80, living in a nursing home, or deceased were excluded. All cases (*n* = 519) were identified, and a random selection (*n* = 254) was performed by CAQ staff. Based on our project budget, we aimed to recruit 100 women with an expected response of 40% based on CAQ staff experiences of recruiting cancer patients employing the same recruitment approach. Therefore, a random selection of 254 cases (using a random number function in Structured Query Language) was approached to participate. The Cancer Register provides an accurate sampling frame for recruitment of a population-based sample as there is a legal requirement that all cancer diagnoses (except non-melanoma skin cancers) are notified to the Register.

### Procedures

CAQ staff mailed potential participants study materials with a reply-paid envelope. Non-responders were sent up to two reminders. Written consent and completed questionnaires were returned to CAQ, scanned and forwarded to the study investigators using a secure file transfer. At the end of the questionnaire, participants were invited to participate in an interview to provide a more detailed account of their cancer journey.

### The research team

Our team includes researchers with content expertise in cancer epidemiology, health psychology, qualitative research, information needs in people affected by cancer, and health inequities. Interviews were conducted with a female qualitative researcher trained in psychology. Three consumers with a lived experience of gynecological cancer (including one consumer with a lived experience of endometrial cancer) guided development of the self-administered questionnaire.

### Questionnaire

The self-administered questionnaire obtained information on sociodemographic (i.e., age, months since diagnosis, marital status, education, private health insurance, country of birth) and cancer-related factors (i.e., cancer history, events leading to diagnosis, treatment) and was completed at one point in time. Participants were asked to think back to the times before diagnosis, during diagnosis, and during treatment (i.e., touchpoints). The following tools were used to measure patient experiences at each of the key touchpoints.

*Need for information* was collected using a study-specific measure for each touchpoint: pre-diagnosis (11 items), during diagnosis (10 items), during treatment (12 items). This was designed using six items from an existing measure (i.e., Supportive Care Needs Survey-Short Form, SCNS-SF34) [23] and in consultation with consumers. Participants rated their level of need for each item on a 5-point Likert scale based on the SCNS-SF34 (from 1 = not applicable to 5 = high unmet need) [23]. *Satisfaction with cancer information*, as measured by the Satisfaction with Cancer Information Profile (SCIP-B), measures satisfaction with the type and timing of information, including seven items rated on a 5-point Likert scale (from 1 = very dissatisfied to 5 = very satisfied) and totaled with a possible score ranging from 7 (very dissatisfied) to 35 (very satisfied) [24]. Two overall ratings for satisfaction and helpfulness of the information were added based on consumer feedback. Participants were asked to select, for each touchpoint, the *top three preferred sources of receiving information* (as measured by HINTS survey items) [25].

Consumers tested the questionnaire and provided feedback over the telephone with one of the investigators (TD). Changes were made to non-validated questions and these were thought to improve the comprehensibility (e.g., wording and clarity of questions, order of questions), relevance (e.g., survey items and response options), acceptability (e.g., ability and willingness to answer questions), and feasibility (e.g., the amount of time taken to complete the survey) of the questionnaire. The consumer advisors indicated their satisfaction with the changes made.

### Qualitative interviews

Semi-structured telephone interviews were scheduled at participants’ convenience, with only the interviewer (BW) and the participant present. Interviews were audio-recorded and transcribed verbatim by a professional transcription service. Interviews continued until data saturation was reached, defined as sufficient richness and recurring patterns in data without new directions or questions [26]. A journey map and topic guide supported notetaking and ensured coverage of experiences across touchpoints, including information needs and experiences (Supplementary Material S1). While the quantitative survey was focused on pre-diagnosis, diagnosis, and treatment touchpoints, the journey map also included post-treatment as a touchpoint. Interviews were participant-directed as much as possible to afford women the space to tell their story [27]. Hence, participants also discussed post-treatment information experiences, and these data were included in the analysis.

### Data analysis

Quantitative, descriptive data analysis was performed with Stata [28]. The characteristics of the cohort contacted, non-participants, participants, and interview participants were summarized as counts with proportions for categorical variables and medians with minimums and maximums for continuous variables. Where too few participants were available for reporting (*n* < 5), an approximate proportion is provided to maintain participant anonymity. Summary statistics for information variables are presented for each key touchpoint.

Individual information need items were dichotomized into no-to-low (1 to 3) and moderate-to-high (4 to 5) unmet needs (as per the scoring for the SCNS-SF34) [23]. The prevalence of unmet needs at each touchpoint is presented with items ranked from highest to lowest based on prevalence. In line with the SCIP-B guidelines, mean values of completed items were imputed for missing items in cases where at least four of the seven items were completed [24]. Total SCIP-B scores were then summarized with means and standard deviations (SD). Responses to individual SCIP-B items and the two overall items were dichotomized as satisfied (4 to 5) and dissatisfied (1 to 3) with prevalence reported at each touchpoint. Responses for each of the preferred sources of receiving information were summed to identify the most prevalent preferences at each touchpoint.

The purpose of the qualitative analysis was to gain a fuller understanding of patient experiences and to ultimately contextualize the quantitative results. This was conceived of within a critical realist paradigm in which we were attentive to gynecological cancer experiences as both socio-culturally and materially situated [29]. To achieve this purpose, we applied content analysis, a flexible and descriptive method especially applicable where existing theory and evidence are limited [30]. Following Hsieh and Shannon’s guidelines [30], we applied both a deductive (directed) approach, using information domains (needs, satisfaction, sources) from the quantitative questionnaire to structure an initial codebook, and an inductive (conventional) approach, allowing findings to emerge without predefined codes.

Three investigators (TD, BW, AD) independently analyzed a random selection of the interviews (8 interviews each) and manually coded references to information (needs, satisfaction, sources). The investigators met several times during this process to discuss coding and emergent findings and to support a consensus process in terms of the credibility of the analysis.

## Results

### Sample

Of the 254 women who were contacted, 82 returned a completed questionnaire (32% response). Questionnaires were completed between November 2023 and April 2024. Of those, 35 participants expressed interest in the qualitative interview and 24 were interviewed (two participants were not contactable; one decided not to be interviewed when contacted; eight expressions of interest were received following completion of interviews). Interviews lasted between 35 and 90 min (median 65 min) and took place between November 2023 and March 2024.

Characteristics of non-participants and participants are presented in Table 1. Demographic characteristics were broadly similar for participants who participated in the study and those who were contacted but did not participate. Further characteristics of all study participants (*n* = 82), and separately for those who did (*n* = 24) and did not (*n* = 58) take part in an interview are presented in Table 2. Study participants were a median of 63 years of age (range 33, 81) and 12 months (range 2, 19) post-diagnosis at the time of completing the survey.

**Table 1:** Characteristics of the study participants and non-participants

Characteristics^a^	Participants (*n* = 82)		Non-participants (*n* = 172)	
	*n*	(%)	*n*	(%)
Age group at diagnosis				
< 50 years	12	(15)	28	(16)
50–69 years	52	(63)	109	(64)
≥ 70 years	18	(22)	35	(20)
Remoteness^b^ of residence at diagnosis				
Major city	56	(68)	94	(55)
Inner regional	18	(22)	51	(30)
Outer regional	np	(< 20)	np	(< 20)
Remote/very remote	np	(< 10)	np	(< 10)
Socio-economic status^c^				
Disadvantaged	25	(31)	np	(< 40)
Middle	47	(57)	103	(60)
Affluent	10	(12)	np	(< 10)
First Nations status				
Non-First Nations peoples	np	(≥ 90)	160	(93)
First Nations peoples	np	(< 10)	12	(7)

^a^Source: Queensland Cancer Register

^b^Remoteness: The relative remoteness of residence at time of diagnosis is derived from the Australian Standard Geographical Classification (ASGC) [20]

^c^Socio-economic status: Based on the Socio-Economic Indexes for Areas (SEIFA) [21]

*np* not publishable (cell count suppressed due to small numbers)

**Table 2:** Characteristics of study participants

Characteristics^a^	Interview participants (*n* = 24)		Non-interview participants (*n* = 58)		All participants (*n* = 82)	
	Median	Min, max	Median	Min, max	Median	Min, max
Age in years at survey	60	(33, 79)	63	(33, 81)	63	(33, 81)
Months since diagnosis	13	(5, 16)	12	(2, 19)	12	(2, 19)
	*n*	(%)^b^	*n*	(%)^b^	*n*	(%)^b^
Marital status						
Married/living together	14	(58)	31	(53)	45	(55)
Not partnered	10	(42)	27	(47)	37	(45)
Education						
High school or less	7	(29)	27	(46)	34	(41)
TAFE/college	6	(25)	15	(26)	21	(26)
University	11	(46)	16	(28)	27	(33)
Private health insurance						
No	7	(29)	28	(50)	35	(44)
Yes	17	(71)	28	(50)	45	(56)
Country of birth						
Australia	19	(79)	48	(83)	67	(82)
Overseas	5	(21)	10	(17)	15	(18)
Prior personal history of cancer						
No	17	(71)	37	(65)	54	(67)
Yes	7	(29)	20	(35)	27	(33)
Disease stage (FIGO) at diagnosis^c^						
I/II	np	(≥ 80)	28	(67)	np	(> 70)
III/IV	np	(< 20)	14	(33)	np	(< 30)
Time since receiving last treatment						
< 6 months ago	np	(< 30)	25	(46)	np	(> 40)
6 or more months ago	np	(≥ 70)	29	(54)	np	(< 60)
Treatment received						
None	np	(< 20)	np	(< 10)	np	(< 10)
Surgery only	8	(35)	25	(44)	33	(41)
Surgery and adjuvant therapy	12	(52)	21	(37)	33	(41)
Adjuvant therapy only	np	(< 10)	np	(< 20)	np	(< 10)

^a^Comparisons between participants and non-participants were made using chi-squared tests for categorical variables and analysis of variance for continuous variables and all *p*-values were > 0.05

^b^Prevalence among non-missing observations (frequency of missing ranges 0 to 17)

^c^Source: Queensland Cancer Register

*min* minimum, *max* maximum, *np* not publishable (cell count suppressed due to small numbers)

Interview participants were similar to non-interview participants and while there were some differences observed, these were not statistically significant (Table 2).

### Unmet information needs

At least one moderate-to-high unmet need was reported by 58%, 71%, and 57% of participants during pre-diagnosis, diagnosis, and treatment, respectively (Table 3). The average number of moderate-to-high unmet needs reported was 3 (range 0–11), 4 (range 0–10), and 3.5 (range 0–12) at each of the respective touchpoints. Proportions ranged by individual need items (17–47% pre-diagnosis, 16–63% during diagnosis, and 15–48% during treatment). The top need during pre-diagnosis, diagnosis, and treatment was “how to treat symptoms/bodily changes you were experiencing” (47%), “being informed about your test results as soon as possible” (63%), and “different types of treatment options/centers” (48%), respectively.

**Table 3:** Prevalence of moderate-to-high unmet information need items during pre-diagnosis, diagnosis, and treatment

Unmet information need item	Rank	*N* (%)^a^
Pre-diagnosis (before you first saw a doctor)		
How to treat symptoms/bodily changes you were experiencing	1	34 (47)
Whether the symptoms/bodily changes you felt are normal or not	2	32 (44)
What action to take	3	30 (43)
When to seek medical advice	4	29 (41)
What questions to ask a doctor	5	25 (36)
Symptoms/bodily changes	6	24 (34)
Which type of doctor to see	7	20 (29)
Causes of cancer/risk factors for cancer	8	18 (26)
Cancer screening, testing, early detection	9	14 (21)
Prevention of cancer	10	12 (17)
Tools to assist with tracking symptoms	11	11 (17)
***% reporting at least one moderate-to-high unmet need***	***-***	***46 (58)***
Diagnosis (during the time you were getting diagnosed)		
Being informed about your test results as soon as possible^b^	1	49 (63)
Which type of cancer you have	2	47 (62)
Where the cancer is in your body (e.g., seeing a diagram)	3	45 (59)
Being given explanations of tests for which you would like explanations^b^	4	43 (56)
How to manage symptoms of cancer	5	40 (53)
Choice about which specialists you see	6	38 (49)
Whether children/family members are at risk	7	25 (34)
Support for family members or carers	8	18 (24)
Accessing professional counselling^b^	9	15 (20)
Available support groups	10	12 (16)
***% reporting at least one moderate-to-high unmet need***	***-***	***56 (71)***
Treatment (during the time you were being treated)		
Different types of treatment options/centers	1	36 (48)
Things you can do to help yourself to get well^b^	2	34 (47)
The benefits and side effects of treatments before you choose to have them^b^	3	35 (46)
The likelihood of a cure for your cancer	4	33 (45)
Important aspects of managing your illness and side effects at home^b^	5	33 (43)
The expected time to recover physically	6	32 (43)
Support for family members or carers	7	14 (19)
Communicating with your workplace	7	14 (19)
The potential loss of fertility and premature menopause	8	13 (18)
Sexual wellbeing	8	13 (18)
Accessing professional counselling^b^	9	12 (16)
Available support groups	10	11 (15)
***% reporting at least one moderate-to-high unmet need***	***-***	***45 (57)***

^a^Prevalence among non-missing observations (frequency of missing ranges 4 to 17); moderate/high unmet information need (rating 4 or 5 on the 5-point Likert scale)

^b^Items from the Supportive Care Needs Survey (SCNS-SF34)^21^

### Satisfaction with information

Approximately 50% (range 45–54%) of women were satisfied/very satisfied with the type and timing of information pre-diagnosis, between 61 and 80% during diagnosis, and between 64 and 80% during treatment (Table 4). The lowest prevalence for satisfaction was for “usefulness of information to your partner/family” and this was consistently the lowest at each touchpoint. Prevalence for overall satisfaction with the amount and helpfulness of information was lowest pre-diagnosis (49% and 52%, respectively), compared to during diagnosis and treatment (> 70% for amount and helpfulness at both touchpoints). Total SCIP-B satisfaction scores were lowest pre-diagnosis (mean 24.6, SD 6.7), and improved during diagnosis (mean 27.5, SD 6.9) and treatment (mean 27.5, SD 6.9).

**Table 4:** Satisfaction with information during pre-diagnosis, diagnosis, and treatment

SCIP-B items and overall	Pre-diagnosis		Diagnosis		Treatment	
	*n*	(%)^a^	*n*	(%)^a^	*n*	(%)^a^
Usefulness of information to you	37	(54)	56	(76)	55	(80)
Usefulness of information to your partner/family	30	(45)	44	(61)	45	(64)
Amount of written information	33	(49)	56	(75)	56	(79)
Amount of verbal information	32	(46)	59	(80)	57	(78)
Timing of which you found information	33	(49)	55	(73)	50	(70)
Amount of detail of the information	31	(46)	54	(72)	52	(73)
How understandable the information was to you	36	(53)	60	(80)	54	(78)
***Total SCIP-B, mean (SD)***^***b***^	Mean	(SD)	Mean	(SD)	Mean	(SD)
	24.6	(6.7)	27.5	(6.9)	27.5	(6.9)
	*n*	(%)^a^	*n*	(%)^a^	*n*	(%)^a^
Overall satisfaction with the amount of information	35	(49)	56	(73)	54	(76)
Overall satisfaction that the information was helpful	35	(52)	58	(76)	60	(85)

^a^Prevalence among non-missing observations (frequency of missing ranges 5 to 15); satisfied/very satisfied with information (rating 4 or 5 on the 5-point Likert scale)

^b^Based on the seven-item SCIP-B scale (range 7 to 35 where higher scores indicate higher satisfaction)^22^

### Preferred sources of receiving information

The most commonly reported preferred sources of information across pre-diagnosis, diagnosis, and treatment included a “doctor or healthcare provider” (70%, 91%, and 86%, respectively), and an “internet search” (63%, 56%, and 29%, respectively) (Table 5). “Family or friends” was among the top three preferred sources pre-diagnosis (29%), and “printed materials” was in the top three preferred sources during diagnosis and treatment (41% and 38%, respectively).

**Table 5:** Women’s preferred sources of receiving information during pre-diagnosis, diagnosis, and treatment (top three shown in bold)

Source of information	Pre-diagnosis		Diagnosis		Treatment	
	*n*	(%)^a^	*n*	(%)^a^	*n*	(%)^a^
Doctor or healthcare provider	**56**	**(70)**	**73**	**(91)**	**69**	**(86)**
Internet search	**50**	**(63)**	**45**	**(56)**	**23**	**(29)**
Family or friends	**23**	**(29)**	15	(19)	13	(16)
Printed materials	17	(21)	**33**	**(41)**	**30**	**(38)**
Cancer organization telephone information and support line	5	(6)	8	(10)	15	(19)
Government health agencies	5	(6)	8	(10)	8	(10)
Complementary or alternative practitioner	7	(9)	7	(9)	5	(6)
Books	6	(8)	5	(6)	5	(6)
Social media	3	(4)	2	(3)	2	(3)
Library	1	(1)	0	(0)	0	(0)
Magazines or newspapers	1	(1)	0	(0)	0	(0)
Support group	0	(0)	1	(1)	0	(0)

^a^Prevalence among participants with at least one response selected (*n* = 2 participants did not select a preferred source pre-diagnosis and diagnosis; *n* = 7 participants had not received treatment and therefore this question during treatment was not answered). Multiple responses were allowed^23^

### Qualitative findings: information needs, satisfaction, and sources

Patient experiences and information (needs, satisfaction, sources) are presented for each key touchpoint, including “post-treatment,” with supporting quotes (Table 6). Direct quotes from participants are provided alongside a participant number, aimed to maintain anonymity and show the diversity of data presented. The qualitative data generally supported quantitative findings. Women wanted more information about symptoms, diagnosis, and treatment. They also highlight that information experiences are complicated, and many participants identified ways in which the quality, frequency, and delivery of information could be improved.

**Table 6:** Illustrative quotations from participants

Themes and subthemes	Illustrative quotes
Pre-diagnosis, before first seeing a doctor	
Recognition of symptoms	I think I noticed symptoms, but the issue was, I didn’t think it was anything abnormal. (P148) I always, for a long time, had a very heavy, painful period. I was always sort of told, “Oh, it’ll go away when you have children”. So, it’s one of those things, you only know what you know. So, for me, it obviously seems normal. (P138) I was about 53 so it’s a couple of years since I had a period, but I thought maybe it was just, you know pre-menopause. I just thought maybe I just hadn’t gone through menopause. (P166) It wasn’t until my dad was diagnosed with something and he was expected to live for four to six months with it. Within about two weeks of being diagnosed, he died. I’m 64 years old and I’d not had a period for a long, long time. I bled for maybe two days. I put it down to emotion at the beginning, but everybody said, “Go see your doctor.” (P149) I started to bleed again, and I had massive pains. But thinking, you know, it’s all the physical work [renovations to the house] that I’m not used to and also maybe the gastric bypass. (P166) I had a tiny light bleed and that’s what caused me to seek medical questions. (P120) The tell-tale signs was the bleeding and that little bit of gel. Like a pink jelly substance. (P145) It was a pain that went with the bleeding. I just needed the pain to stop. (P166)
Motive to seek medical advice	It was lucky, really. So what it is, you know how females chat and females talk, and you remember little snippets. So I remember a friend of mine a couple of years ago, she was definitely menopausal and everything, but she had a little tiny bit of bleeding. She didn’t have the same sort of cancer as me but it was the same sort of thing where she had this little tiny show in her undies when she knew she shouldn’t be having any blood. When I went to the loo, I saw this tiny little bit of pink, just a little five cent piece. So, I acted really quickly when I saw that little bit of pink. It was just that trigger I don’t get a period anymore, I shouldn’t have that, you know? (P116) I went to the toilet and moved my bowels and there was a little smear of blood on the toilet paper. And then the same thing happened the next day. So, I thought, well I should probably check it out. (P140) The bleeding, I thought, was under control for a little while there. And all of the sudden it came back. I thought when I got to this age, it’ll all be gone, but no. (P158) My vision was, I just could not see very well. I ended up with my sister taking me to the emergency. My sister was saying, “You were just so out of it, you were speaking but it was not making any sense at all”. They ended up diagnosing me was I had mini strokes in the brain [which led to the endometrial cancer diagnosis]. (P139) I ended up with a really severe abdominal pain. I would’ve said probably like up to an eight or a nine on the scale and got my husband to take me out to the ED. (P160) It certainly did catch me off guard. I didn’t have any idea. I just noticed I was very busy but quite tired and then I develop, what I call, a little bit of what I thought was a period. I just innocently spoke to [my GP] and said, “Is this normal at my age?” and she flagged it and said, “No, it’s not”. (P105)
Diagnosis, during the diagnostic process	
Taking action	I was lucky, you know. It was a little bleed. I bled for maybe two days. Most doctors would say, “Let’s wait”, but he just jumped straight on it. (P149) I had a blood test and they said, “You’re completely through it [menopause]”. Then I had a good 18 months and then I started getting erratic spotting. I kept going back to the doctor who I’d seen for probably the last 25 years, a male doctor. He just said, “Well, that’s just normal” and I said, “Well, what can I do about it?” and he said, “Nothing, that’s just life, isn’t it? For a female at your age?” I was getting that type of comment all the time. (P145) He [the doctor] said this is quite normal at your age, to get very heavy periods leading up to menopause. (P148) She [the GP] immediately ordered an ultrasound. Then she decided we needed to do a Pap smear and a mammogram and just a basic blood test and a scan. (P105) But the moment I told the female doctor my symptoms she sent me straight away for an ultrasound. My female doctor, I owe my life to her, to be honest. (P145)
The waiting game	It was traumatic, having to wait, it felt like a lifetime. (P171) To wait an extra week till you found out you had cancer, that was a horrible, horrible week for my husband and I. (P149)
Lack of clarity of information	I think one of the best things the gynecologist did was he drew me a picture. He sat there, he drew me the picture. He labeled everything. Like still to this day, year, nearly a year and a half later, I still have that picture. (P157) I must admit, it was by his reaching I knew something was amiss because they kept asking to reposition me. So they kept sort of saying “Can you roll this way” and “We might just take another couple of pictures”. I thought, “Oh, this is not normal for a scan”. (P105) After the tests were done, they referred me to the gynae team but at no point anyone mentioned cancer. No one just comes out and says, “This is a distinct possibility”. On a phone call I asked, “So, on a scale of 1 to 10 how likely is it that this is cancer?” and she said “Oh a 10” and that was how I found out. So that for me was quite shocking. (P160) They did another biopsy as well and said that was clear now but started to talk about endometrial cancer. And that’s why I was surprised when I got the letter, ‘cause I was like, “Is endometrial cancer and uterus cancer the same?”. (P138) You hear a lot about breast cancer, lung, ovarian, brain, leukemia…but you really don’t hear anything about uterine cancer. So, to me there wasn’t a lot of discussion or knowledge out there about uterine cancer. (P139) Endometrial wasn’t one that I really knew a great deal of detail on at all. I certainly hadn’t heard of it prior to my diagnosis. (P173) The gynecologist who was the person who told me and drew me the picture. He was the person who saw me at every appointment. The continuity of care that they provided was so calm. I just had someone I was comfortable with. I didn’t feel nervous to ask questions. I knew what his plan was. I knew everything that was going to happen. I think that just made everything feel like they’ve got it under control. They know what they’re doing. (P157)
Treatment, during the time of planning and receiving cancer treatment	
Information needs about specific aspects of treatment	I think I got the information about the side effects after my first consultation. You can talk to you’re blue in the face, and you can read all the documents, but unless you speak to somebody who’s been through it, you don’t know how bad it can get. (P139) He would just break it down for us. He’d just quickly print out a little fact sheet or my partner could ask him as many questions as he wanted. We were never made to feel silly or like, it was that whole "There's no such thing as a dumb question." (P157) With chemo, you don’t know what to expect. You’ve read everything, but then when you have it, it’s like the theory and the practice, isn’t it? You read the theory, and then you go and have the practice of whatever it is, but it’s a new thing, isn’t it? (P116) Again, very thorough in talking you through what was gonna happen, what the complications were. She’s very thorough, but that sort of emotional side of it, we didn’t really touch on that part of it. (P173) I suppose from my point of view, it’s like these little practical things, just having to deal with the radiation and deal with day-to-day stuff. They’re the sort of things that it’s not really put about in documents. There’s nothing about the practical side of things. (139) I said to my husband, “Can you bring a pillow” because, you know, just having a full hysterectomy, I’d put a pillow between my tummy and the seatbelt. (P166) I wish someone had told me some of these tricks [e.g., metamucil for constipation, pregnancy underwear, bras from cardiac unit]. (140)
Processing and retaining information	You can only handle so much information at a time. It’s kind of gotta be progressive. (P140) Your brain turns off as soon as you hear that cancer word. (P172) Your mind goes a million miles an hour thinking, “What if”? (P132) First when you’re getting all the information your head spins a bit. (P132) When I was going to my oncology appointment they have this little stand of Cancer Council documents, the little pamphlet books. Thankfully, one was endometrial cancer. That was my only sort of reference guide that I had to go on, to just even go and get any sort of confident knowledge from, apart from the Cancer Council website. (P157) One of the things that I find is when you go to the oncologist and they say, “Do you have any questions?”. For me, it’s hard to say; I do have questions because I don’t know what to expect. I don’t know what’s going on. (P139) They say, “knowledge is power”, but sometimes my anxiety, it’s worse for me to know. (P154) It [the trial] has actually been a great source of information for me. You’re filling in their survey and say, “Well, this is what other people are getting and I must be okay ‘cause I haven’t got that one, that one and that one, but I might have that one”. And if you’ve got these questions, other people have got these symptoms. (P140)
Post-treatment	
Aftercare	And then the last appointment. I had to put in a [dilator]. I’m too embarrassed to ring up. (P102) What I’d suggest for future people to have maybe more like a video you can take home in your own privacy to discuss and explain [the dilator]. (P102) The only thing that really caught me off guard was the dilators that you have to use once everything has completed. That was surprising. They waited until the end to tell me that one. That’s a bit evil because they probably knew I wouldn’t have such treatment if I knew that [the dilators were necessary]. (P105) I didn’t want to speak to the radiologist because he’s a guy. I didn’t want to ask him this question [about the dilator]. (P105)
Transition from active treatment to survivorship	I’m not as happy with what’s happened since I finished my treatment. I’m feeling very uncared for. I was the number one patient, I did whatever I was told to do, I did without question. Why am I only have two blood tests in a whole year? Why can’t I just have the CT scan? (P116) When I first saw the oncologist at the end of March, I was a bit surprised. I was expecting every six months I’ll have to have a blood test. And nothing! I was a bit surprised because I know there’s different cancers. A friend of mines has a blood test every six months. It was a sort of very flimsy answer. So when I had the gynecology appointment there was no asking for blood tests. (P102) Now that I’ve finished all of this treatment, why, at the back of my mind, am I to worry, that I’m honing-in on a possibility that it could still be lurking? (P116) I had my clearance in November from the hysterectomy. She [GP, surgeon?] said, “You know your body, just keep an eye out for this and that, you know?” About a week ago I started bleeding a little bit, so I started panicking. Do I just go to a normal GP when that happens or have I gotta get a referral to a gynecologist? (P158) Maybe having somebody to talk to would’ve been nice afterwards; can it come back up again? I’ve not asked any of those questions yet. (P149) My problem was is I had this surgery in [a city] and I had both chemo and radiation up here [in a regional area]. So the surgeon’s trying to give you pamphlets and give you your support groups and everything where they’re all based in [the city]. Then once you sort of chat to the nurses, all of this information was given to me. (P116) I went to my GP and they didn’t know I was cancer free. I got the diagnosis that I was cancer free probably four or five months after I had my last treatment. I thought, “Did the hospital not communicate with the GPs?”. They [GPs] couldn’t even get on to have a look at my bloods or anything like that. (P116)

#### Pre-diagnosis, before first seeing a doctor

Recognition of symptoms:“I think I noticed symptoms, but the issue was, I didn’t think it was anything abnormal.” (P148)

Many participants described uncertainty around whether the symptoms they experienced were normal or not. Heavy and irregular bleeding were perceived as normal by many in the sample (P138) or attributed to other causes including menopausal changes (P166), emotional stress (P149), or increased physical exertion (P166). For some, heavy or irregular bleeding was seen as a “tell-tale sign” (P120) warranting medical attention, including when the bleeding was prolonged or combined with mucous vaginal discharge (P145) or pain (P166). Other symptoms that participants noticed included feeling tired, feeling bloated and loss of appetite.

Motive to seek medical advice:“You know how females chat… I remember a friend of mine… She didn’t have the same sort of cancer as me but it was the same sort of thing where she had this little tiny show… It was just that trigger….” (P116)

Participants described seeing a doctor soon after experiencing a symptom (e.g., P116 pink vaginal discharge; P140 post-menopausal spotting), when a symptom did not resolve as expected (e.g., P158 expected that bleeding would stop as she got older), or presented to the emergency department with blurred vision (P139 mini strokes) or severe abdominal pain (P160). Female friends and loved ones were identified as sources of information about unusual symptoms and what actions to take (P116), whereas for some, their concerns arose opportunistically during healthcare appointments that were made for another reason (P105).

#### Diagnosis, during the diagnostic process

Taking action:“Most doctors would say, let’s wait, but he just jumped straight on it.” (P149)

Women shared varied experiences of having their symptoms acted upon by their healthcare professional. A number of participants described symptoms being dismissed by their healthcare professional as being typical for their age (P145, P148). While some participants expressed gratitude that their healthcare professionals acted immediately (P105, P149), others felt let down by the lack of action (P145). One woman waited two years to be diagnosed citing a male doctor dismissed her post-menopause bleeding and described “I owe my life” to the female general practitioner who helped her get diagnosed (P145).

The waiting game:“It was traumatic, having to wait, it felt like a lifetime.” (P171)

For many participants, their experiences of waiting (or not) for information on their test results and timely communication of their diagnosis were critical to their cancer diagnosis experience. Some described that waiting for diagnostic test results caused significant distress (e.g., P149). We found that these moments of waiting were significant because they involved the most amount of uncertainty. For example, one participant waited ten weeks to see a specialist, which she described as “traumatic” (P171).

Lack of clarity of information:“I think one of the best things the gynecologist did was he drew me a picture.” (P157)

Some only realized that cancer was potentially being investigated due to the behavior of a healthcare professional (e.g., additional images requested during a scan, P105) rather than from being told directly. Some did not understand the purpose of particular tests (noting the potential for incidental cancer test results) and therefore the cancer diagnosis came as a shock (P160). Some felt that the diagnosis itself was not clear, with confusion about the diagnosis being endometrial cancer or uterine cancer (P138). Participants cited a lack of available information about endometrial cancer (e.g., P139) or that they had not heard of endometrial cancer before their diagnosis (e.g., P173). As an example of the importance of clear and respectful communication, one participant described her experience with a doctor who took the time to explain the cancer, including drawing a picture to aid her understanding (P157).

#### Treatment, during the time of planning and receiving cancer treatment

Information needs about specific aspects of treatment: “Unless you speak to somebody who’s been through it, you don’t know how bad it [the side effects of treatment] can get.” (P139)

Experiences with receiving information about treatment and side effects varied among participants. Some described being satisfied with the thoroughness and timeliness of the information they received (P157), while others felt unprepared for treatment despite receiving information (P116). Several noted that information on the emotional impact of treatment was lacking (P173). There was also an information gap addressing practical challenges (“day-to-day stuff,” P139), such as travelling to and from treatment (P166), and appropriate clothing for radiation treatment (P140). Participants also emphasized the need for healthcare professionals to proactively provide information as they did not know what questions to ask (P139), especially for sensitive issues they may be unaware of or find difficult to raise. Some women received information later than they would have liked, and felt it often did not convey the severity of potential side effects (P139).

Processing and retaining information:“You can only handle so much information at a time. It’s kind of gotta be progressive.” (P140)

Some highlighted the mental load of managing their diagnosis and treatment. Participants referred to their “brain turn[ing] off” when they heard the word cancer (P172), their “mind going a million miles an hour” (P132), or that their “head spins” with all the information (P132). Some suggested that information be provided progressively (P140). Participants spoke about various forms of support to help them to remember information from appointments, ask questions, or make treatment decisions. For example, support came from partners, family, friends, health professionals, and specific sources of information (e.g., P157 Cancer Council booklet). Some participants reflected that because they did not know what to expect, they did not know what questions to ask (e.g., P139). For some, more information increased their feelings of anxiety (e.g., P154). One participant found information through a trial on the surgical treatment of endometrial cancer (P140). The trial survey provided a list of treatment-related symptoms which helped normalize potential side effects.

#### Post-treatment

Aftercare: “They waited until the end to tell me that one.” (P105)

Women discussed the lack of information about vaginal dilators, including what was involved in their use and why they were necessary after radiation therapy. For some, this lack of information caused distress and treatment decision regret (P105). Vaginal dilators were a sensitive topic and barriers to information seeking included embarrassment and not wanting to talk to a male doctor (P102, P105). Several participants suggested that videos on how to use dilators would be a useful resource to watch in their own time, as many times as needed, in the privacy of their own home (P102).

Transition from active treatment to survivorship:“I’m not as happy with what’s happened since I finished my treatment. I’m feeling very uncared for.” (P116)

Participants expressed anxiety about finishing active treatment due to reduced frequency of interaction with their healthcare team, inadequate explanations for follow-up frequency, and awareness of different follow-up schedules for other cancer survivors (P102). However, for other participants, frequent follow-ups induced anxiety due to confusion about their risk of recurrence (P116). Fear of cancer recurrence or uncertainty regarding their risk were common (P116). In some cases, this was exacerbated by a lack of information on what signs and symptoms to watch for and who to contact if symptoms appeared (P158).

Having someone to talk to after completing treatment, such as a counsellor, was identified as a needed resource that was currently lacking. While this is not solely related to information needs, such a resource could provide women with tailored information to understand their prognosis and risk of recurrence (P149). One woman suggested support groups and patient advocacy associations as good sources of information and other support, which nurses had directed her to (P116). Additionally, the need for better information sharing and coordination between health services was noted (P116).

## Discussion

This mixed methods study examined information experiences across the cancer continuum among women diagnosed with endometrial cancer. Survey data demonstrated substantial unmet needs and dissatisfaction from pre-diagnosis to treatment, while interviews indicated a need for the amount, timing, and type of information to be tailored to an individual’s needs.

Our findings confirm previous research that unmet information needs are prevalent among people with cancer during and post-treatments [31–34], that many endometrial cancer survivors are dissatisfied with the type and timing of information about diagnosis, treatment and follow-up [17], and that the preferred sources of information consistently include a doctor/healthcare professional, internet search, family/friends, and printed materials [35–37]. The results of our study extend existing evidence on information experiences by including the pre-diagnosis phase, identifying experiences unique to endometrial cancer survivors, and including qualitative interviews to provide context.

To illustrate the importance of pre-diagnosis experiences, the prevalence of reporting at least one moderate-to-high unmet information need at this time was similar to the prevalence during treatment (58% and 57%, respectively). Furthermore, satisfaction with information was lowest pre-diagnosis. The most prevalent unmet information needs pre-diagnosis were about symptoms and what action to take. Some participants described heavy or irregular bleeding as symptoms that were normalized or misattributed to other causes by themselves and instances by healthcare professionals. The only other qualitative study to explore communication pre-diagnosis of endometrial cancer was conducted at one hospital in urban New Zealand, which found that symptom uncertainty contributed to delayed presentation and diagnosis [15].

The poor awareness of endometrial cancer symptoms by participants coupled with the high prevalence of unmet information needs on what action to take highlights the need for accessible information sources. A recent evaluation of Australian online information resources for gynecological cancer symptoms found abnormal/persistent bleeding to be identified in almost three-quarters of resources, although the quality (i.e., readability) and actionability (i.e., identified action steps or advice) of available resources were inadequate [38].

Our results also identified experiences unique to endometrial cancer survivors. For example, two of the most prevalent unmet information needs during diagnosis were about which type of cancer a woman had and where the cancer is in her body (62% and 59%, respectively). Qualitative interviews further explain that some people feel confused about the differences between technical terms used to describe their cancer as uterine cancer or endometrial cancer, which made conversations with healthcare professionals difficult to understand. One woman described this need being met when her doctor drew a picture of where the cancer was in her body.

The lowest prevalence for satisfaction across all touchpoints was for the usefulness of information to their partner/family. The research in the area of gynecological cancer caregivers is limited, even though family are often the main source of (emotional and financial) support, in addition to providing a caregiving role (e.g., treatment monitoring, symptom management, personal care) [39]. While this did not arise specifically in interviews, many participants talked about having someone with them when receiving or interpreting information. Unmet information needs are prevalent among informal caregivers (i.e., partners and family of people affected by any cancer type) during survivorship [40, 41], expressing need for timely and individualized information [40]. Information needs pre-treatment is an understudied area of research [40, 41]. Future research into the unmet information needs across the cancer continuum for partners and family should be prioritized and the integration of support people (e.g. care navigators) into the health system could reduce unmet needs for both patients and caregivers.

Our study has some limitations, particularly the low response. While the distribution of characteristics among participants who completed the quantitative questionnaire is similar to non-participants, our sample consisted of a slightly higher proportion of women living in major cities (although this was not statistically significant). Satisfaction with information was similar across remoteness areas except residents of major city areas reported lower satisfaction during treatment compared with people in regional areas (mean 26.2, standard deviation 7.2 vs 30.4 and 4.9; *p* < 0.05). Therefore, the level of satisfaction reported here may be somewhat overstated. Although this study examined patient experiences along the information pathway from pre-diagnosis through treatment, post-treatment information experiences were not captured in the survey. As a result, the survivorship phase of the cancer care continuum was not systematically investigated. While interview participants were free to discuss experiences beyond treatment, the spontaneous nature for discussing this may have introduced a bias towards negative experiences as survivorship was not explored systematically with all participants. However, experiences were not exclusively negative with a minority of the sample largely describing positive accounts of their survivorship. In addition, the cross-sectional design of the study introduces the potential for recall bias with participants asked to reflect retrospectively on their experiences. Surveys were completed between 2 and 19 months following diagnosis therefore the accuracy of recall may vary across touchpoints and between individuals. Finally, we used a study-specific questionnaire to measure information needs, and while we drew on an existing validated tool, the six items of the SCNS-SF34 that were included have not been validated for use in isolation.

Qualitative findings, which are not intended to be generalizable, provide a fuller description of patient experiences and contextualize the quantitative results. As information needs post-treatment are well-researched compared with earlier phases of the cancer continuum [40], we did not include post-treatment as a touchpoint in the quantitative survey, although participants were not limited by this during qualitative interviews and were free to discuss their experiences.

## Conclusion

This study shows that many women with endometrial cancer encounter significant information gaps across the care continuum, particularly during symptom recognition, diagnosis, treatment decision-making, and post-treatment transition. Even the lowest-ranked needs were reported by one in seven participants, underscoring the breadth of unmet information needs. A flexible, patient-centered communication approach, tailored to people’s preferences, capacities, and timing, is essential. Providing information for family and carers may further enhance support. Training all healthcare providers in open communication techniques could improve patient comfort, reduce barriers to information seeking, and increase satisfaction with care. Future analyses may be able to identify specific recommendations to meet the information needs of this population.

## Supplementary Information

Below is the link to the electronic supplementary material.

ESM 1 Supplementary Material 1 (DOCX 102 KB)

## Data Availability

The authors confirm that the data supporting the findings of this study are available within the article. The questionnaire and data that support findings of this study are available on request from the corresponding author. The data are not publicly available due to privacy and ethical restrictions.

## Footnotes

1 We use the term “women” herein to refer to and include all people born with female reproductive organs.
