# Supplementary material for: Information experiences among women diagnosed with endometrial cancer: a mixed methods study
Source: Support Care Cancer. 2026 Aug 11;34(9):841. doi: 10.1007/s00520-026-11082-2 (PMC13461649; doi:10.1007/s00520-026-11082-2)
Supplement: Supplementary file 1 [file 520_2026_11082_MOESM1_ESM.docx]

**Supplementary Material S1: Interview Guide and Journey Map**

**General questions for the interview:**

- The questionnaire you completed asked about your information needs during your cancer journey. Tell me about your own cancer journey, from early on until now.
- Describe your personal experiences as a series of steps.
- Touchpoints along the cancer journey
  - What were you doing/actions taken during this time?
  - What were you thinking during this time?
  - Who did you speak to during this time?
  - What were you feeling during this time?
- Tell me about your information needs and experiences.
- Tell me about your communication needs and experiences.

**Anything else questions:**

- Is there anything else we haven’t touched on yet that you would like to tell me about?

**Journey map:**
